# Trends in the utilization of psychotropic medications in China from 2018 to 2021

**DOI:** 10.3389/fphar.2022.967826

**Published:** 2022-09-07

**Authors:** Xinyan Zhang, Xiaowen Hu, Yuxuan Zhao, Christine Y. Lu, Xiaoyan Nie, Luwen Shi

**Affiliations:** ^1^ Department of Pharmacy Administration and Clinical Pharmacy, School of Pharmaceutical Sciences, Peking University, Beijing, China; ^2^ Department of Population Medicine, Harvard Medical School and Harvard Pilgrim Health Care Institute, Boston, MA, United States; ^3^ International Research Center for Medicinal Administration, Peking University, Beijing, China

**Keywords:** psychotropic medications, trend, utilization, hospitals, China

## Abstract

**Background:** Monitoring psychotropic medicine consumption trends can provide information on the extent of pharmacological interventions for mental disorders and availability of psychotropic medicines.

**Objectives:** This study aimed to illustrate the trends in psychotropic drug utilization in China’s hospitals.

**Methods:** We retrospectively analyzed the aggregated monthly psychotropic procurement records of 1009 hospitals from 31 provinces in China from January 2018 to September 2021. Total psychotropic medicine consumption included the sales of antipsychotics, antidepressants, anxiolytics, mood stabilizers, and sedatives or hypnotics. Information, including generic name, procurement amount, dosage form, strength, purchase time, and geographical data, was collected. Population-weighted psychotropic utilization was expressed in defined daily dose per 1000 inhabitants per day (DDD/1000/day).

**Results:** Psychotropic medicine sales increased from 4.5 DDD/1000/day in Q1 2018 to 6.4 DDD/1000/day in Q3 2021; total utilization in China’s hospitals increased by 42.2%. The use of each class of psychotropics showed a gradually increasing trend. Antidepressants were the most consumed psychotropics, accounting for 48.4% of the total psychotropic utilization (3.1/6.4 DDD/1000/day), followed by sedatives or hypnotics (31.3%; 2.0/6.4 DDD/1000/day) and antipsychotics (15.6%; 1.0/6.7 DDD/1000/day). Among all sub-classes of psychotropics, a most significant growth in DDD per 1000 inhabitants per day was seen for selective serotonin reuptake inhibitors (1.2–1.9 DDD/1000/day), whereas the consumption of typical antipsychotics (from 0.1 to 0.09 DDD/1000/day) and tricyclic antidepressants (from 0.05 to 0.03 DDD/1000/day) decreased during the study period. Psychotropic utilization substantially increased between Q1 2018 and Q3 2021 in regions with different economic levels. In Q3 2021, total psychotropic utilization in secondary and tertiary hospitals was 9.4 DDD/1000/day and 6.0 DDD/1000/day, respectively. Sedatives or hypnotics in secondary hospitals accounted for the largest proportion of utilized psychotropics (43.6%; 4.1/9.4 DDD/1000/day), whereas antidepressants were the most commonly used psychotropic in tertiary hospitals (50.0%, 3.0/6.0 DDD/1000/day).

**Conclusion:** This study showed that despite increases in psychotropic medication use, the consumption of medicines is still much lower than in other countries and regions internationally. With reference to the estimated prevalence of corresponding mental disorders, our study illustrates that a large treatment gap for mental health problems exists in China. In addition, the wide use of psychotropics with weak clinical evidence raises serious concerns regarding rational use. Greater efforts are needed to increase the availability of psychotropic medicines and to facilitate proper psychotropic use.

## Introduction

Globally, an estimated 700 million people have a mental disorder, and the prevalence of various mental health problems continues to rise (Colton and Manderscheid, 2006; Whiteford et al., 2013). In recent decades, mental disorders have continued to be the chief contributor to the global burden of disease and the sixth leading cause of disability-adjusted life-years (DALYs) (GBD 2017 DALYs and HALE Collaborators, 2018), posing a serious challenge to health systems. However, the health systems of countries across the world have not yet adequately responded to the burden of mental disorders, and there is often a substantial gap between the need for mental health treatment and its availability (WHO, 2013). The WHO has developed many initiatives and interventions to promote the comprehensive health of people worldwide (Chokshi et al., 2016; Keynejad et al., 2021), such as the development of the WHO Comprehensive Mental Health Action Plan 2013–2030 (WHO, 2013) and the inclusion of mental health in the Sustainable Development Goals (Saxena et al., 2014; Chokshi et al., 2016).

Psychotropic medications are effective and often recommended as a first-line treatment in countries to manage mental symptoms (Cipriani et al., 2018). These medications can be subdivided into multiple categories including antidepressants, antipsychotics, mood stabilizers, sedatives, and hypnotics. Systematic monitoring of psychotropic consumption trends can provide information on the extent of pharmacological interventions for mental disorders and can also be used to inform future policy evaluations (WHO, 2013). Results from recent studies reporting psychotropic use ranged from 0.9 to 249.1 DDD per 1000 inhabitants per day across different countries and regions worldwide, with total consumption corresponding to a 4.1% relative average increase annually (Brauer et al., 2021). This growth, mainly in antidepressants and antipsychotics, may suggest the improved accessibility and acceptability of mental health treatment; however, it also raises concerns about more prevalent polypharmacy and increasing off-label use. Previous research found that off-label use in up to 51% of patients using second-generation antipsychotics (Driessen et al., 2016).

Moreover, access to psychotropic medicines among individuals with mental disorders remains insufficient in some countries where resources are scarce. Almost 85% of people with severe mental disorders receive no treatment with medicines for their disorder (WHO, 2013), and only 16.5% of those who do receive treatment received minimally adequate treatment (Thornicroft et al., 2017).

China accounts for approximately 20% of global DALYs attributable to mental disorders, ranking first in the world (Charlson et al., 2016; WHO, 2018). The lifetime prevalence of any mental disorders is 16.6% in the general population, and mental disorders have become more common across China (Huang et al., 2019). However, due to the relative shortage of mental health resources and services in China and other low- and middle-income countries, mental disorders were frequently undertreated. In China, individuals are more likely to deny psychological conditions that may have experienced in the past compared to individuals from other cultures (Phillips et al., 2007; Phillips et al., 2009), because of culturally sensitive questions as well as the patients’ deep-rooted stigma in society (Liang et al., 2018). In addition, the lack of health workers with the appropriate authority to prescribe medications means that the prescription of psychotropics relies heavily on doctors in mental health specialties and in psychiatric departments of general hospitals. In 2015, China had approximately 1.5 psychiatrists and 1.7 psychiatric beds per 100,000 population (Wang and Tian., 2018), while in 2011, upper-middle-income countries had approximately 2.0 psychiatrists and 2.7 psychiatric beds per 100,000 population. Therefore, the use of psychotropic drugs has further been restricted. In recent years, the Chinese government has strengthened mental health policies and programs to improve the access and quality of services for people with mental illness and has begun to monitor the widespread use of psychotropics.

The number of psychotropics available on the market has gradually increased, providing a great variety of treatment options for prescribers (González-López et al., 2015; Luo et al., 2020). The cost of different psychotropic medicines varies considerably in China (Yu et al., 2020); for example, the average annual cost of escitalopram and duloxetine was higher than that of other antidepressants, while tricyclic antidepressants (TCA) were associated with the lowest annual average cost (USD10.62) (Ding et al., 2022). Although previous surveys showed that the consumption of antidepressants increased annually in local areas of China (Gao et al., 2013), with all psychotropic drugs consumed at 4.57 DDD per 1000 inhabitants per day in 2019 (Brauer et al., 2021), the generalizability of these findings was limited by the focus on a single class of psychotropic drugs and limitations pertaining to their study timeliness. To date, little is known about the latest complete information on the consumption of psychotropic medicines in China. To fill this important knowledge gap and better inform clinical practices regarding the rational use of psychotropics, this empirical study aimed to describe the consumption trends of psychotropic medicines in China.

## Methods

### Study design

We retrospectively analyzed the quarterly trends in psychotropic drugs procurement in 1009 hospitals from 31 provinces in China from January 2018 to September 2021.

### Data sources

We used procurement data from the electronic database of China Medical Economic Information (CMEI), a large government-approved information database, and service platforms covering the records of public hospitals in mainland China. The database, which was constructed in 1993, captures sample institutes from 396 secondary public-sector hospitals and 763 tertiary hospitals. The secondary hospitals are regional hospitals that provide health services across several communities. Tertiary hospitals provide health services across regions, provinces, cities and to the whole country, and are the highest level of hospitals. The procurement records of participating hospitals accounted for approximately 40% of total drug procurement at city level public hospitals in China (China Medical Economic Information Network, 2022). Public hospital pharmacies account for over 80 percent of the market share of psychotropic drugs in China. Therefore procurement records in the CMEI were provided as acquisition data for participating hospitals. The hospitals were sampled hierarchically based on their geographical and economic levels. We selected hospitals on the basis that they each had full psychotropic procurement records during the entire study period. Based on this criterion, the selected 718 tertiary hospitals represented 49.6% of all tertiary hospitals and the selected 291 secondary hospitals accounted for 6.7% of all secondary hospitals in the study regions (Table 1).

**TABLE 1:** Distribution of sample hospitals.

Economic levels^a^	Tertiary	Secondary
Most developed	131/225 (58.2%)	44/331 (13.3%)
Developed	171/394 (43.4%)	84/818 (10.3%)
Upper-middle developed	118/234 (50.4%)	42/803 (5.2%)
Lower-middle developed	91/203 (44.8%)	44/713 (6.2%)
Primary developed	45/145 (31.0%)	31/590 (5.3%)
Underdeveloped	162/248 (65.3%)	46/1107 (4.2%)
Total	718/1449 (49.6%)	291/4362 (6.7%)

a Classification of the regions was obtained from the China Health Statistics Yearbook. Most developed region: Beijing, Tianjin, Shanghai, Jiangsu, and Fujian; Developed region: Zhejiang, Shandong, Hubei, Chongqing, and Guangdong. Upper-middle developed region: Inner Mongolia, Shanxi, Anhui, Liaoning, and Hunan; Lower-middle developed region: Sichuan, Jiangxi, Henan, Hainan, and Ningxia; Primary developed region: Xinjiang, Xizang, Yunnan, Qinghai, and Jilin; Underdeveloped region: Shaanxi, Hebei, Guizhou, Guangxi, Heilongjiang, and Gansu.

### Data collection and management

We extracted the monthly psychotropic drug purchase data from the CMEI electronic database. Information including the generic name, Anatomical Therapeutic Chemical code, procurement amount, dosage form, strength, purchase time, and geographical data were collected. Psychotropic medications were categorized into five major medicine classes according to the Anatomical Therapeutic Chemical (ATC) Classification System maintained at the recommendation of the WHO Collaborating Center for Drugs Statistics Methodology (WHO Collaborating Centre for Drug Statistics Methodology, 2022), including antipsychotics (N05A), antidepressants (N06A), anxiolytics (N05B excl. N05BA), mood stabilizers (N03A and N05A), and sedatives or hypnotics (N05BA, N05CD, and N05CF). Each class was subdivided mainly according to the mode of action into different subclasses; the number of drugs in each class and subclass is shown in Supplementary Table S1. We excluded ADHD medications as they are mainly used in children and adolescents, which make direct comparisons with other psychotropic medicines inappropriate. Data were managed and analyzed using Microsoft Excel 2019 and STATA 15.0 (StataCorp LLC, Texas, United States).

### Data analysis

To obtain procurement data for simple comparisons of drug utilization across countries and across different formulations of the drug, the data were converted into the defined daily dose per 1000 inhabitants per day (DDD/1000/day). The DDD corresponds to the average maintenance dose per day of the drug when used for its main indication in adults. We adopted the following equation to calculate the weighted population as a proxy for the population covered by our sample hospitals.

$$Y_{i}=\sum_{i=1}^{31}p_{i}\times \frac{n_{i}}{N_{i}}\times \frac{m_{i}}{M_{i}}$$

$Y_{i}$ : Coverage inhabitants in a given year

$P_{i}$ : Total population in a given year in province i

$n_{i}$ : Number of sample hospitals in province i

$N_{i}$ : Number of total hospitals in province i

$m_{i}$ : Number of inpatients and outpatients in the sample hospitals in province i

$M_{i}$ : Number of inpatients and outpatients in all hospitals in province i

This equation was based on the following two assumptions. The first assumption was that there was no significant difference in the distribution of sample hospitals across provinces. The second assumption was that there was no significant difference in the distribution of the population covered by sample hospitals across provinces. To ensure accuracy in calculating inhabitants, the inhabitants’ coverage was calculated for outpatients and inpatients. The inhabitants’ coverage was calculated separately for secondary and tertiary hospitals and at different economic levels. The classification of the economic levels based on the rank of 2018 provincial per capita gross domestic product (GDP) among the provinces studied was provided by the ***China Health Statistical Yearbook*** and the relevant census data were collected from the ***China Statistics Yearbook***.

## Results

### Trends in total psychotropic utilization in China’s hospitals

**FIGURE 1:**
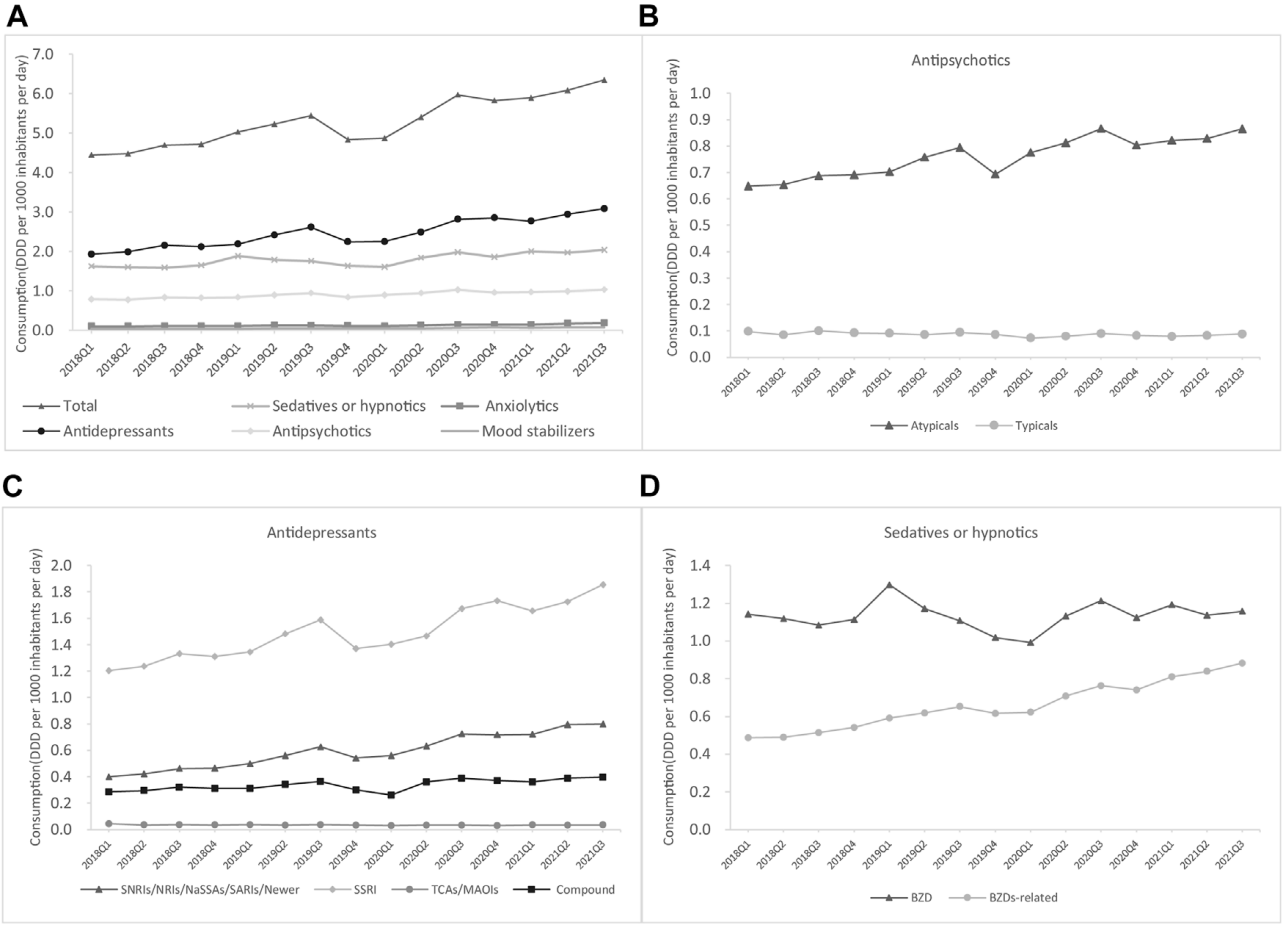
Trends in psychotropic utilization for each class in China’s hospitals **(A)** Overall **(B)** Antipsychotics **(C)** Antidepressants **(D)** Sedatives or hypnotics.

The total consumption of psychotropics in China’s hospitals rose from 4.5 DDD/1000/day in Q1 2018 to 6.4 DDD/1000/day in Q3 2021, representing a 42.2% growth. The utilization of each class of psychotropics between Q1 2018 and Q3 2021 all showed gradual increasing trends, with the most growth seen in antidepressants (from 1.9 to 3.1 DDD/1000/day), sedatives or hypnotics (from 1.6 to 2.0 DDD/1000/day), and antipsychotics (from 0.8 to 1.0 DDD/1000/day) (Figure 1A). Antidepressants were the most consumed psychotropics, accounting for 48.4% of the total psychotropic utilization in Q3 2021 (3.1/6.4 DDD/1000/day), followed by sedatives or hypnotics (31.3%; 2.0/6.4 DDD/1000/day) and antipsychotics (15.6%; 1.0/6.7 DDD/1000/day); anxiolytics (0.2/6.7 DDD/1000/day) and mood stabilizers (0.08/6.7 DDD/1000/day) accounted for a relatively small proportion of the utilized psychotropics. The percentage of each class of psychotropic medication was stable during the study period.

The consumption of psychotropic drugs by class is shown in Figure 1. Among sedatives or hypnotics, the consumption of benzodiazepines (BZDs), which rose from 1.1 to 1.2 DDD/1000/day, was higher than that of BZD-related drugs, which rose from 0.5 to 0.9 DDD/1000/day (Figure 1D). Atypical antipsychotics were the most commonly consumed type of antipsychotics and showed an increase from 0.6 to 0.9 DDD/1000/day (Figure 1B). Selective serotonin reuptake inhibitors (SSRIs) were the most commonly consumed antidepressant and also showed the greatest increase from 1.2 to 1.9 DDD/1000/day, followed by Selective norepinephrine reuptake inhibitors/noradrenaline reuptake inhibitors/noradrenergic and specific serotonergic antidepressants/newer antidepressants (from 0.4 to 0.8 DDD/1000/day) and compound antidepressants (from 0.3 to 0.4 DDD/1000/day), whereas the consumption of typical antipsychotics (from 0.1 to 0.09 DDD/1000/day) and TCA (from 0.05 to 0.03 DDD/1000/day) decreased during the study period (Figure 1C).

**FIGURE 2:**
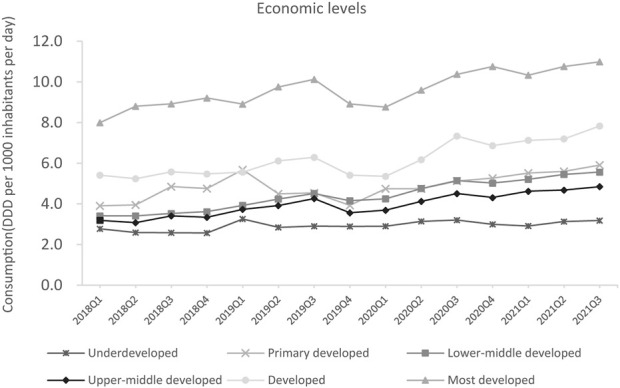
Psychotropic utilization in different economic levels.

Psychotropic utilization substantially increased between Q1 2018 and Q3 2021 in regions with different economic levels. The most developed regions and developed regions were the top two largest consumers of psychotropics in Q3 2021, with 7.8 and 11.0 DDD/1000/day, respectively. The consumption of psychotropic medicines in underdeveloped regions was relatively low (3.2 DDD/1000/day) (Figure 2).

### Psychotropic utilization in secondary and tertiary hospitals

**FIGURE 3:**
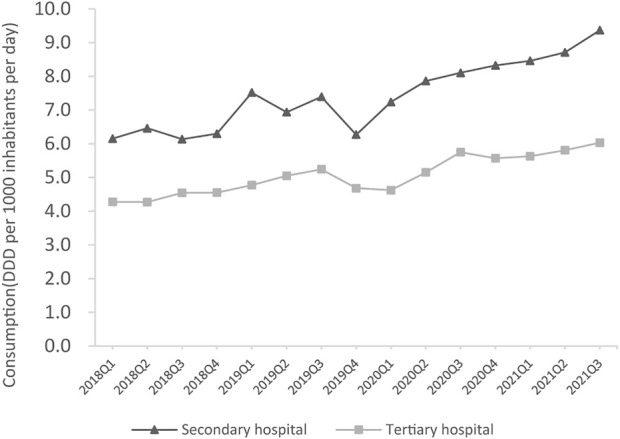
Overall psychotropic utilization in secondary and tertiary hospitals.

**FIGURE 4:**
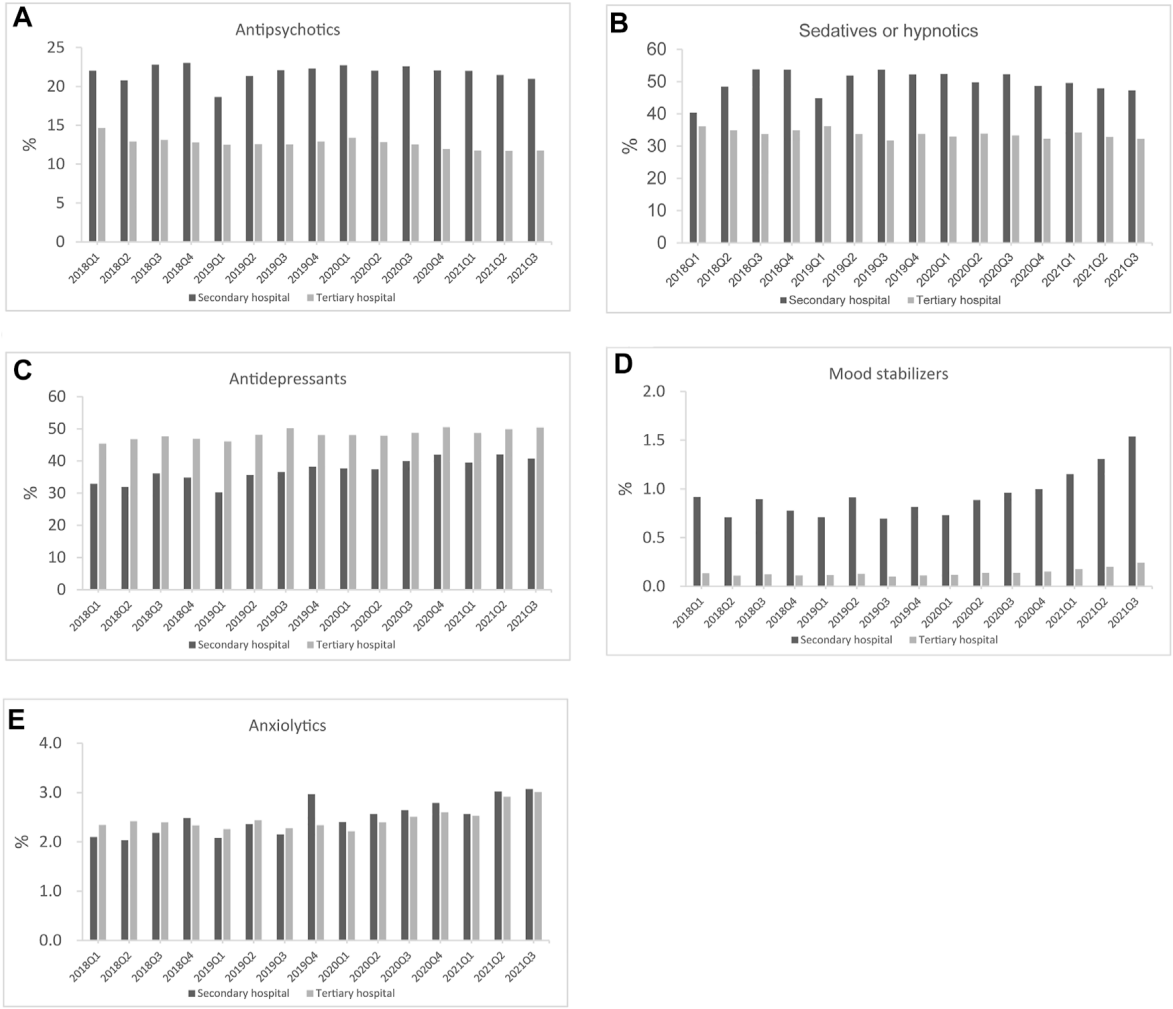
Psychotropic utilization for each class in secondary and tertiary hospitals **(A)** Antipsychotics **(B)** Sedatives or hypnotics **(C)** Antidepressants **(D)** Mood stabilizers **(E)** Anxiolytics.

**FIGURE 5:**
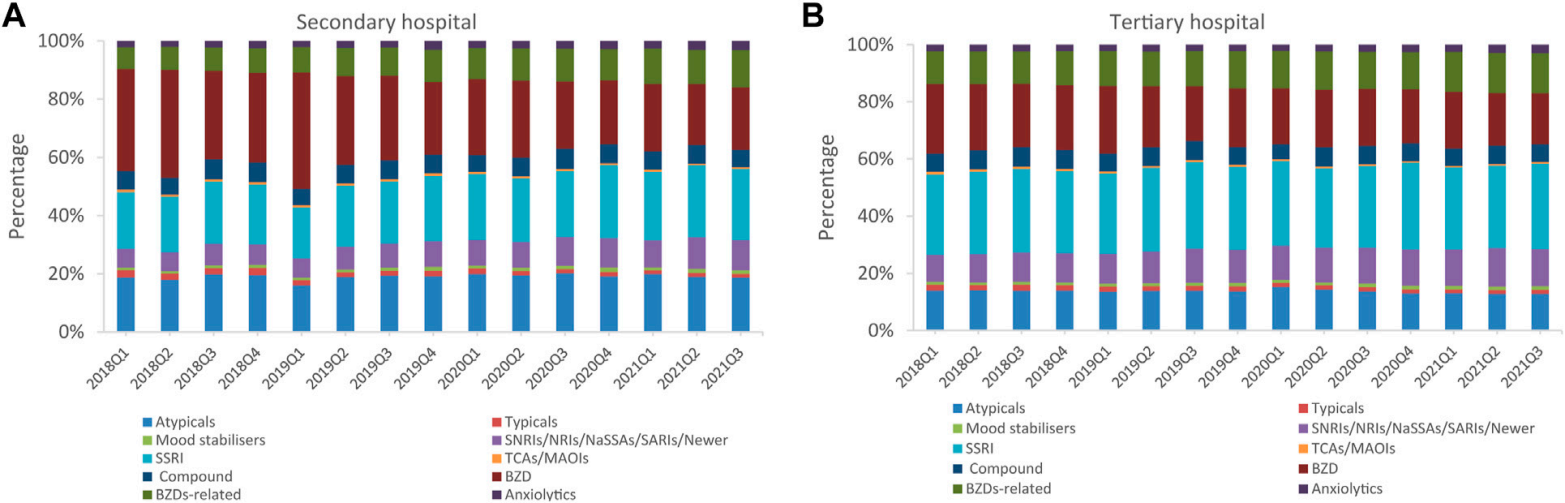
Psychotropic utilization in each subclass **(A)** Secondary hospitals **(B)** Tertiary hospitals.

The total psychotropic utilization in secondary and tertiary hospitals increased by 51.6% (from 6.2 to 9.4 DDD/1000/day) and 39.5% (from 4.3 to 6.0 DDD/1000/day) between Q1 2018 and Q3 2021, respectively (Figure 3). In Q3 2021, total psychotropic utilization in secondary and tertiary hospitals was 9.4 and 6.0 DDD/1000/day, respectively. Sedatives and hypnotics accounted for a higher proportion than antidepressants in secondary hospitals, at 47.8% (4.5/9.4, DDD/1000/day) and 34.0% (3.2/9.4, DDD/1000/day), respectively (Figure 4). In contrast, antidepressants accounted for the highest proportion in tertiary hospitals (50.0%; 3.0/6.0 DDD/1000/day), followed by antipsychotics, which accounted for 19.1% (1.8/9.4 DDD/1000/day), and 13.3% (0.8/6.0 DDD/1000/day) of the total amount in secondary and tertiary hospitals, respectively. Mood stabilizers were the least commonly utilized psychotropic in secondary and tertiary hospitals (1.5% and 0.2%, respectively). For each subclass of psychotropics, BZDs and SSRIs accounted for the largest proportion in secondary and tertiary hospitals, respectively (Figure 5).

**FIGURE 6:**
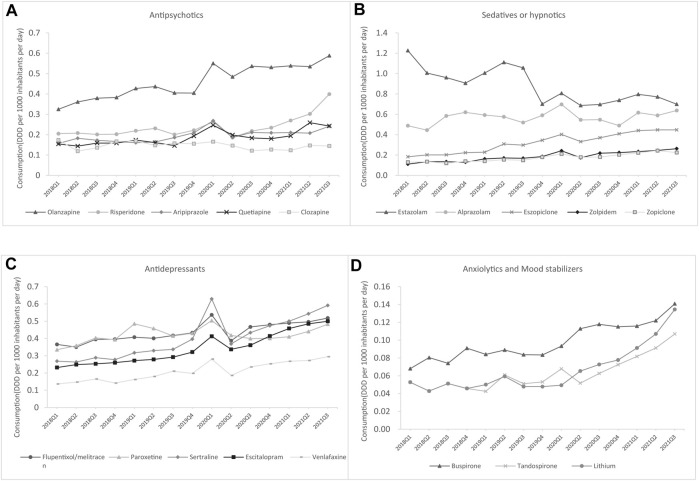
The top five drugs consumed by generic name for each class in secondary hospitals **(A)** Antipsychotics **(B)** Antidepressants **(C)** Sedatives or hypnotics **(D)** Anxiolytics and mood stabilizers.

**FIGURE 7:**
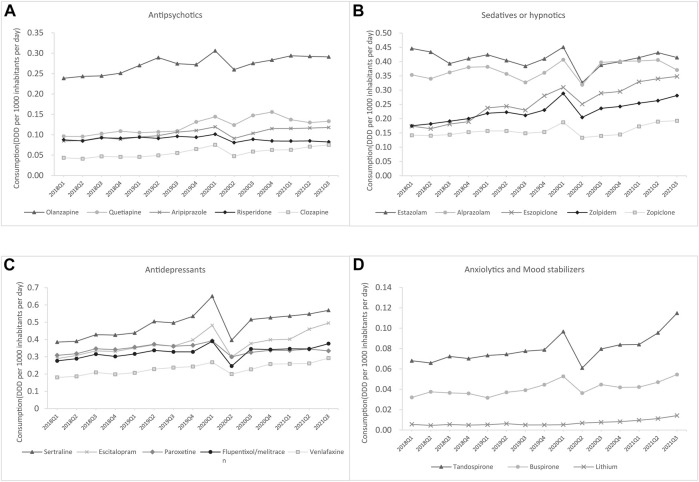
Top five drugs consumed by generic name for each class in secondary hospitals **(A)** Antipsychotics **(B)** Antidepressants **(C)** Sedatives or hypnotics **(D)** Anxiolytics and mood stabilizers.

As shown in Figure 6, estazolam, olanzapine, tandospirone, lithium, and sertraline were the most commonly used medications in tertiary hospitals from each of these five groups, whereas the most commonly used antidepressant in secondary hospitals was flupentixol/melitracen, and buspirone was the most frequently used mood stabilizer until 2021 (Figure 7).

## Discussion

This study is the first to estimate population-based psychotropic consumption at the national level in China using ATC/DDD methodology for international comparison. Our study analyzed psychotropic utilization in China’s hospital setting and benchmarks it internationally, which would inform healthcare providers, decision-makers, and the public. This study found that there was a striking 42.2% increase in psychotropic dispensing from Q1 2018 to Q3 2021, but the consumption of psychotropic medicines in China was still significantly lower than European and American countries and regions, and the observed increases in total psychotropic DDD/1000/day were largely driven by increases in antidepressants (from 1.9 to 3.1 DDD/1000/day).

In this study, we found that the total consumption of psychotropic utilization gradually increased from 4.5 DDD/1000/day to 6.4 DDD/1000/day in China between Q1 2018 and Q3 2021. This trend is comparable with other psychotropic sales studies in America, Canada, Portugal, the UK, France (Brauer et al., 2021), and Austria (Stephenson et al., 2013). The increase in the use of psychotropic medicine suggests more awareness of mental health as a pivotal part of overall health (Prince et al., 2012), and the growing acceptance of psychotropic drug use (Lewer et al., 2015). However, a longitudinal study that reported the consumption of psychotropic medicines in 65 countries and regions found that the consumption of psychotropic medicines in China remains at the lowest levels among countries worldwide (Brauer et al., 2021), which confirms our findings. Disparities in psychotropic medicine consumption of different countries are probably due to multiple factors, such as variations in the role of health technology assessment, service organization and delivery, and medicine reimbursement policies. Our results indicate that the consumption of psychotropics is only sufficient for six patients per 1000 inhabitants per day, as a previous report showed that the 12-months prevalence of any psychiatric disorders in the Chinese population was relatively high (9.3%) (Huang et al., 2019), could implying that the majority of people with severe mental disorders do not have access to treatment with medicines for their disorder or have poor adherence. Moreover, multiple factors, such as higher public stigma, financial or cultural considerations, and poorer recognition of mental illness, might also explain the low consumption (Zito et al., 2008; Wong et al., 2017). Thus, there is an urgent need to improve the potentially undertreatment of patients with psychiatric disorders, especially in developing countries such as China. These governments should focus on increasing the availability of psychotropic medicines by increasing country level health expenditure on mental illness, by introducing psychotropic medicine reimbursement policies, as well as by training health-care professionals to prescribe psychotropic medicines. In addition, future studies are warranted to explore the potential treatment gap between lower- and middle-income countries and high-income countries and regions, in order to further develop strategies for improving mental health.

We observed that the total DDD/1000/day rose for all types of psychotropic drugs and was largely driven by the increase in antidepressant utilization. This may be attributable to a series of policies adopted by the Chinese government to enhance depression prevention and treatment, and the increased public recognition and awareness of depression (Wong et al., 2017; Yu et al., 2020). The results of the psychotropic medicine subclass analyses show that the consumption of SSRIs is rising, and their sales were twice as high as the consumption of all other antidepressants. This trend aligns with the main clinical practice guidelines, which recommend SSRIs for people with moderate-to-severe depression as a first-line antidepressant and as a first-line treatment for generalized anxiety disorders, panic attacks, and post-traumatic stress disorder (NICE, 2018). Moreover, sedative or hypnotic consumption ranked second and consumption levels rose throughout our study, which differs from the findings of some previous studies (Stephenson et al., 2013; Barczyk et al., 2020). This finding might be related to the high use of these medicines for the treatment of insomnia (Doi et al., 2000). Nevertheless, it is noteworthy that there are risks of addiction, dependence, and withdrawal symptoms following long-term use of BZDs (Rickels et al., 1990), and BZDs are not approved for insomnia. Despite these known side effects and misuse, BZDs continue to be widely used. This study shows that the increase in the consumption of overall antipsychotics can be linked to expanded regulatory approval for indications outside psychosis and an increase in their off-label use (Alexander et al., 2011). Mood stabilizer use has increased slightly over the period of investigation, suggesting that mood stabilizers are clinically useful for a growing number of indications, including bipolar disorder, epilepsy, and neuropathic pain (Mackey, 2010).

In this study, psychotropic utilization was greater in most developed regions than in underdeveloped regions. This was possibly because in China, the most developed or developed regions have higher personal disposable income per capita and more adequate health resources and services than underdeveloped regions (Zhang et al., 2017; China, 2019). Therefore, the psychosocial care capacity in more developed regions was better, people with mental disorders needed for mental health care can be met in a more timely manner, and patients have more access to psychotropic medication (Knapp et al., 2006; Liang et al., 2018). Moreover, patients with mental disorders in China also prefer to seek mental treatment in more developed areas, such as Beijing, Shanghai, and Guangzhou, owing to the severity of the disease and inadequate healthcare resources in underdeveloped regions. More importantly, the prevalence of mental disorders is relatively high in developed regions (Natali et al., 2018; Xu et al., 2021), and higher treatment rates lead to higher psychotropic use.

In addition to economic level, we found significant differences in the consumption patterns of psychotropic medicines between secondary and tertiary hospitals. Consistent with previous studies (Stephenson et al., 2013), antidepressants (SSRIs) were dominant in tertiary hospitals, while sedative-hypnotics (BZDs) were consumed more than antidepressants in secondary hospitals. Possible explanations for the much larger increases in psychotropic DDD/1000/day, especially BZDs use, in secondary hospitals compared to tertiary may include the lower cost of medicines, lower income levels of patients, and healthcare professionals’ prescription habits. Thus, there is a strong potential for the inappropriate use of BZDs. Surprisingly, flupentixol/melitracen in secondary hospitals held the leading position in the antidepressant market. As a mixture of TCA and a classical antipsychotic, some studies have suggested that flupentixol/melitracen is associated with significant improvement in quality of life, independent of the presence of anxiety or depression (Hashash et al., 2008). However, there is limited evidence to support the use of this combination. Moreover, TCAs have more side effects and a greater possibility of drug-drug interactions (Qin et al., 2014). Thus, special concerns for safety and rational use should be raised regarding the wide use of this antidepressant with weak clinical evidence (Solanki and Banwari, 2016).

This study has several limitations. First, the hospitals in the database were on a voluntary basis instead of mandatory participation, especially the proportion of secondary hospitals in the study was relatively low; therefore could bring selection bias. Furthermore, the sample hospitals in our study only included all public hospitals, and the sample size was relatively small, but we believe the findings would still be sufficiently representative, as the number of patient visits in public hospitals exceeded 85% of total patient visits in China (China, 2019), and the CMEI database covered 1009 hospitals from 31 provinces of mainland China. Second, we did not have access to bed day data and hospital size, and the population denominator used was determined under certain conditions; therefore, the estimation of the population covered by the sample hospitals might be biased. Finally, our analyses were based on procurement data and did not reflect individual-level treatment for mental health problems. In addition, individual-level claim or prescription data are needed to inform us about potential overuse, misuse, and access to psychotropic medication.

## Conclusion

This study showed that despite increases in psychotropic medication use, the consumption of medicines is still much lower than in other countries and regions internationally. With reference to the estimated prevalence of corresponding mental disorders, our study illustrates that a large treatment gap in mental health problems exists in China. In addition, the wide use of psychotropics with weak clinical evidence raises serious concerns regarding rational use. Greater efforts are needed to increase the availability of psychotropic medicines and facilitate proper psychotropic use.

## Data Availability

The raw data supporting the conclusions of this article will be made available by the authors, without undue reservation.
