# Supplementary material for: Trends in the utilization of psychotropic medications in China from 2018 to 2021
Source: Front Pharmacol. 2022 Sep 7;13:967826. doi: 10.3389/fphar.2022.967826 (PMC9490132; doi:10.3389/fphar.2022.967826)
Supplement: Supplementary file 1 [file Table1.docx]

**Supplementary Table SI. Psychotropic medications studied according to class and subclass**

| Class | Subclass | Generic Name | Anatomical Therapeutic Chemical code | Number |
| --- | --- | --- | --- | --- |
| Antipsychotics | Typicals |  |  | 16 |
|  |  | Perphenazine | N05AB |  |
|  |  | Fluphenazine Decanoate | N05AB |  |
|  |  | Droperidol | N05AD |  |
|  |  | Haloperidol | N05AD |  |
|  |  | Tiapride | N05AL |  |
|  |  | Thioridazine | N05AC |  |
|  |  | Loxapine | N05AH |  |
|  |  | Chlorpromazine | N05AA |  |
|  |  | Clopenthixol | N05AF |  |
|  |  | Chlorprothixene | N05AF |  |
|  |  | Pipotiazine | N05AC |  |
|  |  | Trifluoperazine | N05AB |  |
|  |  | Sulpiride | N05AL |  |
|  |  | Sultopride | N05AL |  |
|  |  | Penfluridol | N05AG |  |
|  |  | Zuclopenthixol | N05AF |  |
|  | Atypicals |  |  | 9 |
|  |  | Aripiprazole | N05AX |  |
|  |  | Amisulpride | N05AL |  |
|  |  | Olanzapine | N05AH |  |
|  |  | Quetiapine | N05AH |  |
|  |  | Risperidone | N05AX |  |
|  |  | Clozapine | N05AH |  |
|  |  | Paliperidone | N05AX |  |
|  |  | Perospirone | N05AX |  |
|  |  | Ziprasidone | N05AE |  |
| Mood stabilisers |  |  |  | 1 |
|  |  | Lithium | N05AN |  |
| Antidepressants | SSRIs |  |  | 6 |
|  |  | Escitalopram | N06AB |  |
|  |  | Fluvoxamine | N06AB |  |
|  |  | Fluoxetine | N06AB |  |
|  |  | Paroxetine | N06AB |  |
|  |  | Sertraline | N06AB |  |
|  |  | Citalopram | N06AB |  |
|  | SNRIs/NRIs/NaSSAs/SARIs/Newer |  |  | 9 |
|  |  | Agomelatine | N06AX |  |
|  |  | Bupropion | N06AX |  |
|  |  | Duloxetine | N06AX |  |
|  |  | Mirtazapine | N06AX |  |
|  |  | Milnacipran | N06AX |  |
|  |  | Trazodone | N06AX |  |
|  |  | Reboxetine | N06AX |  |
|  |  | Tianeptine | N06AX |  |
|  |  | Venlafaxine | N06AX |  |
|  | TCAs/MAOIs |  |  | 7 |
|  |  | Amitriptyline | N06AA |  |
|  |  | Imipramine | N06AA |  |
|  |  | Doxepin | N06AA |  |
|  |  | Clomipramine | N06AA |  |
|  |  | Maprotiline | N06AA |  |
|  |  | Moclobemide | N06AG |  |
|  |  | Mianserin | N06AX |  |
|  | Compound |  |  | 1 |
|  |  | Flupentixol+melitracen | N06CA |  |
| Sedatives or hypnotics | BZDs |  |  | 10 |
|  |  | Alprazolam | N05BA |  |
|  |  | Estazolam | N05CD |  |
|  |  | Oxazepam | N05BA |  |
|  |  | Diazepam | N05BA |  |
|  |  | Flurazepam | N05CD |  |
|  |  | Lorazepam | N05BA |  |
|  |  | Chlordiazepoxide | N05BA |  |
|  |  | Midazolam | N05CD |  |
|  |  | Triazolam | N05CD |  |
|  |  | Nitrazepam | N05CD |  |
|  | BZDs-related |  |  | 4 |
|  |  | Eszopiclone | N05CF |  |
|  |  | Zaleplon | N05CF |  |
|  |  | Zopiclone | N05CF |  |
|  |  | Zolpidem | N05CF |  |
| Anxiolytics |  |  |  | 2 |
|  |  | Buspirone | N05BE |  |
|  |  | Tandospirone | N05BE |  |
| **Total** |  |  |  | **65** |

*Abbreviations: SSRIs, selective serotonin reuptake inhibitors; SNRIs, serotonin noradrenaline reuptake inhibitors; NRIs, noradrenaline reuptake inhibitors; NaSSAs, noradrenergic and specific serotonergic antidepressants. SARIs,* *selective antagonists reuptake inhibitors; TCAs, Tricyclic antidepressants; MAOIs, monoamine oxidase inhibitors; BZD, benzodiazepines*
